# Lung nodule detection and potential impact on guideline-based management: a retrospective post-market evaluation of three commercial software systems

**DOI:** 10.1007/s00330-026-12702-5

**Published:** 2026-06-24

**Authors:** Anna Jöbstl, Anna K. Luger, Bernhard Nilica, Florian Kocher, Thomas Sonnweber, Ivan Tancevski, Florian Augustin, Laurenz Nagl, Daniel Leitner, Gerlig Widmann

**Affiliations:** 1 Department of Radiology, Medical University of Innsbruck, Innsbruck, Austria; 2 Department of Nuclear Medicine, Medical University of Innsbruck, Innsbruck, Austria; 3 Department of Internal Medicine V, Medical University of Innsbruck, Innsbruck, Austria; 4 Department of Internal Medicine II, Medical University of Innsbruck, Innsbruck, Austria; 5 Department of Visceral, Transplant and Thoracic Surgery, Center of Operative Medicine, Medical University of Innsbruck, Innsbruck, Austria

**Keywords:** Computer aided detection, Lung nodule, Management recommendation, Computed tomography

## Abstract

**Objectives:**

To evaluate three commercial AI software tools for pulmonary nodule detection and segmentation and to assess their impact on guideline-based management recommendations.

**Materials and methods:**

A total of 740 CT and PET-CT studies from clinical routine were analyzed using three software tools (S1, S2, S3). We compared the total number of detected nodules and “actionable” nodules (per British Thoracic Society (BTS) definition). We further evaluated how measurement variations between tools affected hypothetical management according to Fleischner Society and BTS guidelines for incidental nodules.

**Results:**

The tools differed significantly in the total number of detections (S1: 1336; S2: 1060; S3: 1536; *p* < 0.001) and wrong findings (S1: 965; S2: 720; S3: 1169; *p* < 0.001). However, the detection of actionable nodules was comparable across all tools (S1: 375; S2: 341; S3: 373; *p* = 0.73). While no statistically significant differences were found in mean diameter or volume measurements, small absolute variations led to significant differences in management. Specifically, S2 triggered significantly more 1-year follow-up recommendations than S3 under BTS guidelines (*p* < 0.001). No significant management differences were observed when applying Fleischner Society guidelines.

**Conclusion:**

While the three included AI tools show comparable performance in detecting actionable nodules, minor measurement variations significantly impact downstream management when using guidelines with narrow thresholds, such as the BTS criteria. Fleischner Society guidelines appear more robust to these inter-software variations.

**Key Points:**

***Question*** *How do commercial software tools for pulmonary nodule detection perform in real-world settings and impact hypothetical management under BTS and Fleischner guidelines?*

***Findings*** *Detection of actionable nodules was comparable across all tools, but small absolute measurement variations triggered significantly more 1-year follow-up recommendations under BTS guidelines.*

***Clinical relevance*** *AI software can cause inconsistent BTS-based management due to narrow thresholds, while Fleischner criteria appear more stable. Frequent detection of benign lesions potentially poses a risk of overdiagnosis and overtreatment in standalone AI-based reporting.*

**Graphical Abstract:**

**Figure:**
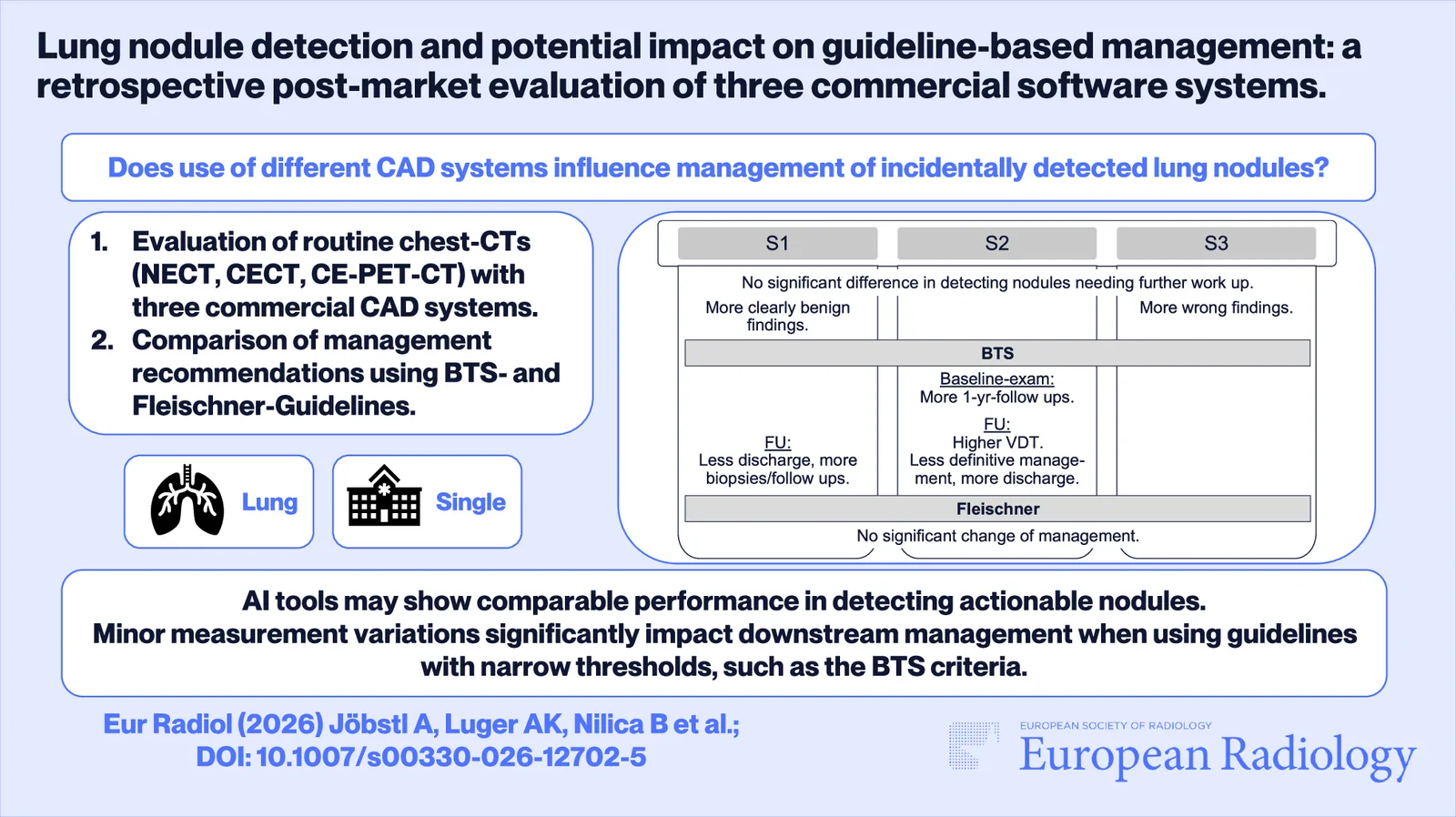

## Introduction

Lung nodules are defined as round or irregular opacifications within the lung parenchyma between 3 mm and 3 cm in diameter [1]. The prevalence ranges between 13% in routine imaging and 33% in screening populations [1]. Solid nodules are more frequently encountered than part-solid or nonsolid nodules; however, they are generally associated with a lower risk of malignancy [2].

An increasing use of CT scans leads to a growing workload for radiologists. To enhance reading efficiency and reduce diagnostic errors, computer-aided detection (CAD) software capable of detecting and segmenting lung nodules is frequently adopted. However, post-market studies evaluating the clinical performance and impact of various CAD tools on nodule management remain scarce. In clinical practice, not only is the detection of individual nodules relevant, but also the assessment of the number of nodules identified per patient [3]. Moreover, the quality of segmentation and the classification into solid or subsolid lesions significantly influence potential subsequent management decisions [1, 3].

The primary objective of this study was to compare the number of detected lung nodules across different commercially available AI software applications and to subsequently assess the number of actionable nodules according to the British Thoracic Society definition of true lung nodules. As a secondary objective, we explored the potential consequences if the classified actionable nodules were incidental findings for which the Fleischner Society and British Thoracic Society guidelines differ (Table 1), and whether this could lead to differences in hypothetical downstream management depending on the AI software used. Lung-RADS was not applied, as its use is restricted to lung cancer screening populations [4].

**Table 1:** A comparative overview of the Fleischner and BTS guidelines with respect to application and key criteria

Criterion	Fleischner Society (2017)	British Thoracic Society (2015)
Target population	Adults ≥ 35 years, incidentally detected nodules, no immunosuppression, no oncologic disease	All adults with incidentally detected nodules
Risk stratification	Low-risk vs. high-risk (e.g., smoking history, age, cancer history)	Quantitative risk model (e.g., Brock model)
Threshold for follow-up	Solid nodules < 6 mm: usually no follow-up	Solid nodules < 5 mm or < 80mm^3^: no follow-up
Measurement method	Diameter-based (mm); Average of long and short axis. Volumetry optional	Volume-based (mm^3^) preferred; Long axis diameter as alternative
Part-solid nodules	Separate recommendations	Separate recommendations
Follow-up intervals	3–24 months, depending on risk profile and size	3–24 months, depending on volume and growth; automated volumetry preferred
Management of growing nodules	Consider biopsy, PET-CT, or resection for significant growth	Risk-based escalation: PET-CT, biopsy, or multidisciplinary discussion
Use of software tools	Mentioned, no explicit recommendation	Recommends the use of dedicated volumetry software

## Materials and methods

This retrospective study was conducted in accordance with the Declaration of Helsinki and approved by the local institutional ethics committee (No. 1040/2024). From clinical routine in a tertiary hospital, patients with thoracic CT scans were consecutively selected from the radiology information system (RIS) and picture archiving and communication system (PACS) (DeepUnity Diagnost 2.0.2.2, Dedalus Healthcare Systems Group) between 01/2023 and 12/2024.

Contrast-enhanced (CECT) and non-contrast-enhanced (NECT) CT scans, as well as contrast-enhanced PET-CTs (CE-PET-CT) of the thorax or including the whole thorax, had been acquired using routine diagnostic protocols in breathhold from five different CT (SOMATOM Drive, SOMATOM Definition Flash, and SOMATOM Definition AS [all Siemens Healthineers]) and PET-CT scanners (Discovery 690 and Discovery MI [both GE Healthcare]).

As we wanted to address the real-life cohort from clinical routine in our hospital, our only exclusion criterion regarding patients was an age < 18 years; furthermore, we only included examinations with an axial lung kernel: slice thickness of 1 mm for CTs and of 1.25 mm for PET-CTs. From the PET-CTs, only the CT-part was viewed; the PET-images were not considered. Any indication was included.

Three commercially available CE-certified software tools for the detection of lung nodules were evaluated: Software 1 (S1): AI-Rad Companion, PULMO VA30A (Siemens Healthineers), S2: contextflow ADVANCE Chest CT-2.1.0 (contextflow GmbH), and S3: Veolity LungCAD 2.1.0. (MeVis Medical Solutions). Each 1 mm/1.25 mm lung window (lung kernel) was exported automatically or manually to all of the three tools.

All software tools were evaluated using the respective vendor-defined default settings. Analyses of prototype configurations with variable sensitivities or in-depth technical validation, including phantom-based segmentation accuracy, were not within the scope of this study.

Examinations, which were declined by at least one of the software tools used, had to be excluded to ensure comparability of the results.

**Fig. 1:**
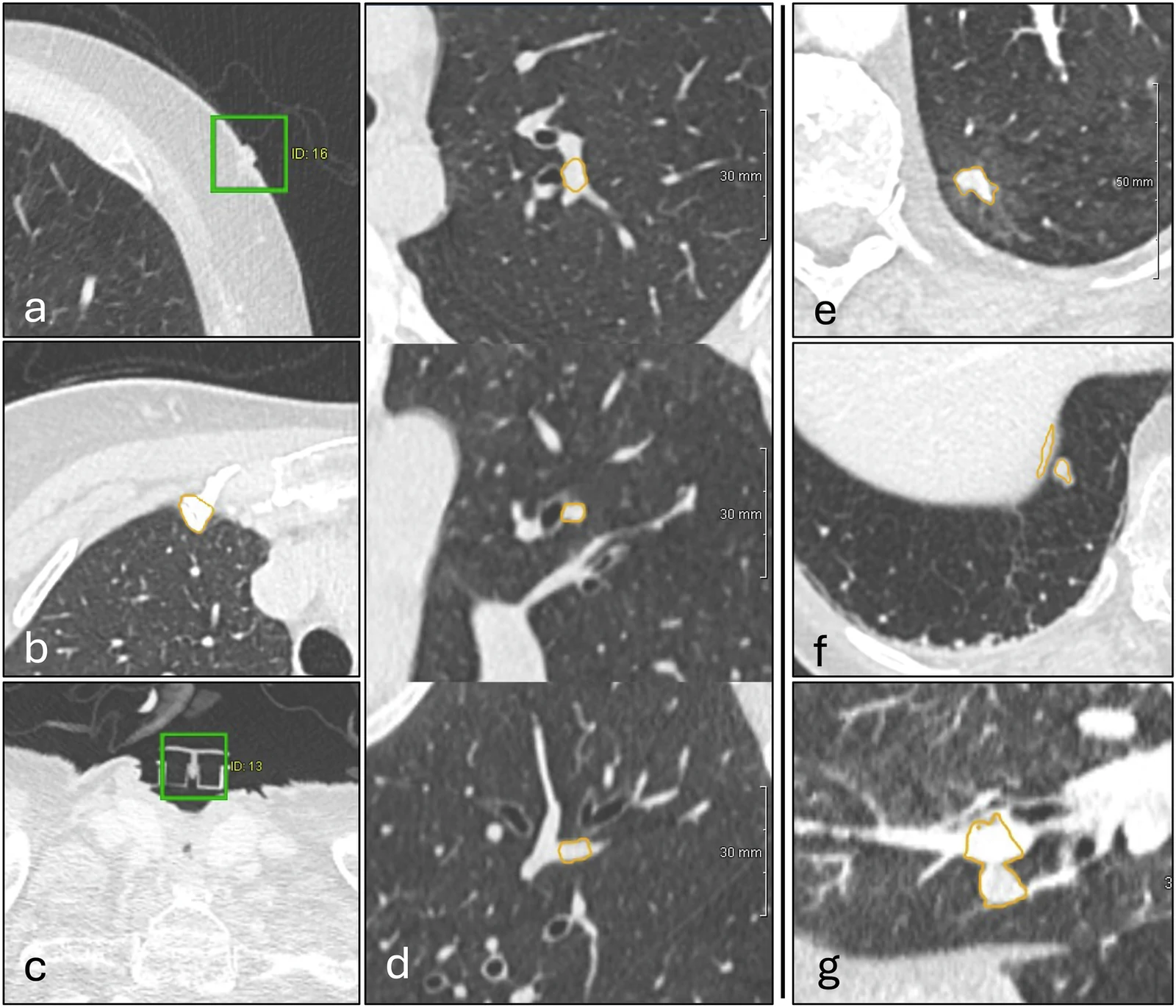
Examples for segmentation: left: “wrong” nodules—**a** mamilla, **b** partial volume of a rib, **c** tracheostomy tube, **d** vessel in axial, coronal, and sagittal slices; right: segmentation accuracy: **e** excellent, **f** poor, **g** failure

Only the software-detected lung nodules between 5 mm and 3 cm in average diameter were reviewed by a board-certified radiologist with experience of > 5 years. Additionally, a subset of randomly chosen nodules was reviewed by a thoracic radiologist with experience of > 15 years to exclude relevant inter-reader-variability. Each nodule was defined as “actionable” (not clearly benign, needing work-up) if it was not “clearly benign” (intrapulmonary lymph nodes, calcified nodules as defined by the BTS definition [1]) or “wrong” (e.g., vessels, partial volumes, extrathoracic structures) (Fig. 1a–d). Intrapulmonary lymph nodes are defined as smaller than 1 cm, perifissural or subpleural within 1 cm of a fissure or pleural surface, and lentiform or triangular in shape with smooth margins. Benign calcified nodules show diffuse, central, laminated, or popcorn-like calcifications [1].

As this study did not aim to compare human versus AI sensitivity, AI results were not blinded. Furthermore, blinding regarding the software vendors was not feasible, as evaluations were performed directly within the respective proprietary interfaces.

The quality of the automated segmentation of the actionable lung nodules was evaluated visually according to Hoop and coworkers and defined as “excellent” (contour perfectly consistent with the nodule), “satisfactory” (contour deviates ≤ 20%), “poor” (contour deviates > 20%) or “failure” (inconsistent or failed segmentation) (Fig. 1e–g) [5]. The available version of S2 did not outline the segmentation, which allowed quality evaluation only for S1 and S3. S3 allows manual correction of the detection and segmentation of nodules, but to maintain comparability, this function was not used.

When available and processed successfully by the respective tools, follow‑up scans were uploaded to S1, S2, and S3 to evaluate potential inter‑software differences in volume doubling time (VDT). Only studies acquired on the same scanners and reconstructed with identical parameters (lung kernel; 1 mm for CT and 1.25 mm for PET‑CT) were included. For VDT calculation, S1 automatically used soft kernel images instead of lung kernel images, a vendor-specific setting that could not be modified.

The tools automatically ranked the detected nodules: long axis diameter by S1 and S2, whereas S3 additionally calculated a malignancy score and used both the long axis diameter and the malignancy score for nodule ranking. For each patient, the five most suspicious, actionable nodules were further evaluated using both the Fleischner and the BTS guidelines. Relevant clinical information, such as nicotine abuse and patient or family history of lung cancer, was obtained from hospital records. For the Brock model, the online tool on the BTS webpage (https://www.brit-thoracic.org.uk/quality-improvement/guidelines/pulmonary-nodules/pn-risk-calculator/) was used. As earlier examinations were not used, each study could be assumed to be the first per patient.

We also analyzed whether any actionable nodules were detected exclusively by one or two of the tools but missed by another. We did not compare the amount of nodules detected by a human reader.

Statistical analyses were performed using Microsoft Excel (for Mac, Version 16.99.1, Microsoft Corp.) and Jamovi (Version 2.6.26.0). For statistical testing, ANOVAs (Welch, Fisher, Kruskal–Wallis) and Chi-squared/Fisher’s exact tests were used. If necessary, post hoc tests (Games–Howell, Bonferroni) were applied. Inter-reader variability for nodule actionability was assessed using the Kruskal–Wallis test, as the data followed a non-normal distribution. For nodule segmentation, Fisher’s exact test was employed to account for the small sample size. Statistical significance was defined as *p* < 0.05.

A priori power analysis was performed using G*Power (version 3.1.9.6, Heinrich-Heine-University) to determine the required sample size for comparing nodule size. Assuming a clinically relevant difference of 2 mm between tools (effect size 0.5, α = 0.05, power = 0.90), a minimum sample size of 86 nodules per tool was required for two-sided *t*-tests. Post hoc power analyses for ANOVA (actionable nodules, effect size 0.3) and tests (Fleischner and BTS guidelines; effect size 0.3) demonstrated high statistical power (0.98 to 1.0).

For inter-reader variability, an a priori approximation using one-way ANOVA (two groups, effect size 0.25, α = 0.05, power = 0.95) indicated a required sample size of 210 nodules. Our actual sample of 265 nodules yielded a post hoc power of 0.98.

## Results

### Study population/patients’ characteristics

**Fig. 2:**
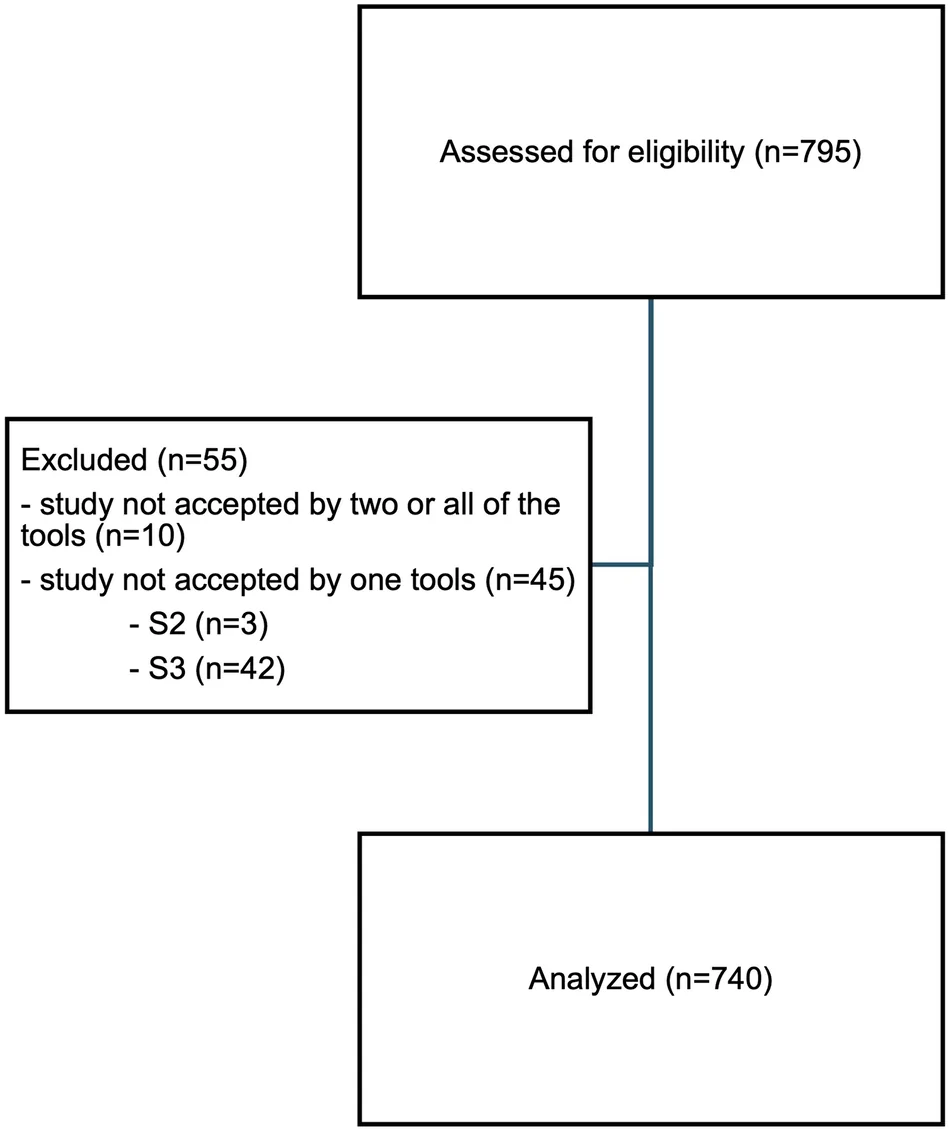
Consort diagram/study flowchart

From the original 795 examinations (= patients), ten were not accepted by two or all of the tools, 45 examinations were not accepted by one tool—from those, 7% (*n* = 3) were declined by S2 and 93% (*n* = 42) by S3. No examination was declined only by S1 (Fig. 2). Specific information regarding the reason for the decline was not provided by the software tools. DICOM non-conformance, reconstruction issues, and slice thickness could be ruled out.

740 patients (398 male (53.8%), 342 female (46.2%)) out of a total of 795 patients could be included in the final analysis. For 110 (S2) and 118 (S1, S3) of those patients, additional follow-up scans were available.

Patients were 19–95 years old, with a mean ± SD of 64.4 ± 13.8 years. Among 516 patients (69.7%), at least one malignancy was documented, most commonly lung and breast cancer. An additional 47 patients (6.4%) had different conditions potentially associated with pulmonary nodules, most commonly rheumatoid arthritis or sarcoidosis. Current or former nicotine abuse was reported in 269 patients (36.4%), while smoking history was not known for the remaining patients due to incomplete clinical documentation. A personal history of lung cancer was present in 124 patients (16.8%), and a positive family history was noted in 14 patients (1.9%).

We analyzed 165 (22.3%) NECTs, 388 (52.4%) CECTs, and 187 (25.3%) CTs from CE-PET-CTs. Indications were “staging” in 423 (57.2%) studies and “routine” (including, e.g., rule out of pulmonary artery embolism (PAE), infection, or interstitial lung disease) in 317 (42.8%) studies (Table 2).

**Table 2:** Patients’ characteristics

No. of patients	740
Male	398 (53.8%)
Female	342 (46.2%)
At least one malignancy	516 (69.7%)
Other diseases with potential lung nodules (e.g., rheumatoid arthritis, sarcoidosis)	47 (6.4%)
Known current or former nicotine abuse	269 (36.4%)
Lung cancer history	
Personal	124 (16.8%)
Family	14 (1.9%)
Modality	
NECT	165 (22.3%)
CECT	388 (52.4%)
CE-PET-CT	187 (25.3%)
Indication	
Staging	423 (57.2%)
Routine	317 (42.8%)

### Inter-reader variability

In a subset of 265 software-detected nodules (S1 35.1%, S2 26.4%, and S3 38.5%) from 50 examinations (= 50 patients) two radiologists (reader 1 (R1)—board-certified with more than 5 years of experience and reader 2 (R2)—board-certified thoracic radiologist with experience of > 15 years) both independently defined 112 (42.3%) nodules as actionable. A total of 16 nodules (6%) were assessed differently, which was not statistically significant (*p* = 0.89).

Segmentation quality was evaluated for S1 and S3 in a subset of 24 nodules (limited to five nodules per patient). Readers scored the segmentation as ‘excellent’ (1), ‘satisfactory’ (2), or ‘poor’ (3); no cases of ‘failure’ (4) were observed. Mean scores for S1 were 1.4 ± 0.71 (R1) and 1.6 ± 0.65 (R2) (*p* = 0.45), while S3 scores were 1.4 ± 0.78 (R1) and 1.5 ± 0.78 (R2) (*p* = 0.75). No statistically significant inter-reader differences were found for either tool.

### Lung nodule detection rate

**Fig. 3:**
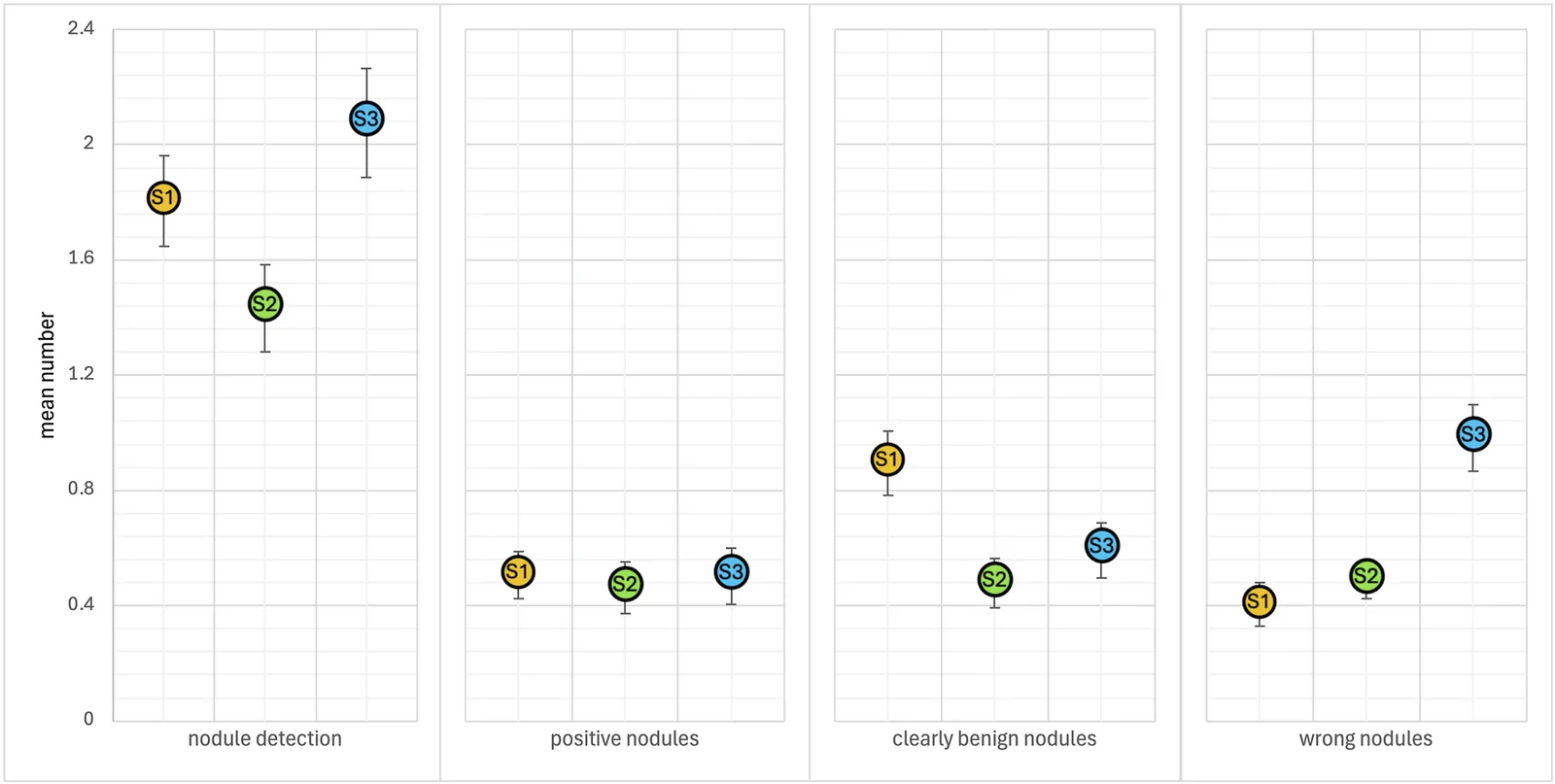
Comparison of means and 95% confidence intervals (CI) of nodule detection per case for S1, S2, and S3

The three software tools showed significant differences in the total number and classification of detected nodules with 1336 (S1), 1060 (S2), and 1536 (S3), respectively (*p* < 0.001). While S3 detected the highest overall number of nodules, it also produced the most wrong findings (730 [47.5%] vs. 303 [22.7%, S1] and 365 [34.4%, S1]) (S3 vs. S1/S2: *p* < 0.001). S1 identified the most clearly benign nodules (662 [49.6%, S1] vs. 355 [33.5%, S2] and 439 [28.6%, S3]) (S1 vs. S2/S3: *p* < 0.001), whereas detection rates of actionable nodules were comparable across all tools (375 [28.1%, S1], 341 [32.3%, S2] and 373 [24.3%, S3]), with no statistically significant differences (Fig. 3).

A total of 102 actionable nodules were not detected by at least one of the three software tools. S2 missed a significant amount of actionable nodules detected by S1 and/or S3 (*p* < 0.001), whereas S1 showed the lowest rate of missed nodules (*p* < 0.001). Most of the missed nodules were solid; however, S1 missed significantly more nonsolid nodules, which were found by the other tools (*p* < 0.001), resulting in a statistically significant difference in density (*p* = 0.02), but not in nodule size (*p* = 0.41). Between 40% and 57% of missed nodules across the tools had a calculated malignancy risk > 10% (Brock model), underlining their potential clinical relevance. Results are summarized in Table 3.

**Table 3:** Comparison of detected lung nodules between S1, S2, and S3

	S1	S2	S3	Comparison
Number of patients	740	740	740	
Detected nodules	1336	1060	1536	*p* < 0.001 S1 > S2: *p* = 0.002 S3 > S2: *p* < 0.001 S1 vs. S3: *p* = 0.08
Nodules per patient	1.8	1.4	2.1	
At least one nodule in (%) of patients	491/740 (66.4%)	444/740 (60%)	516/740 (69.7%)	
Actionable nodules	375/1336 (28.1%)	341/1060 (32.3%)	373/1536 (24.3%)	*p* = 0.73
Actionable nodules per patient	0.51	0.46	0.50	
At least one actionable nodule in (%) of patients	209/740 (28.2%)	186/740 (25.1%)	195/740 (26.4%)	
Clearly benign nodules	662/1336 (49.6%)	355/1060 (33.5%)	439/1536 (28.6%)	*p* < 0.001 S1 > S2: *p* < 0.001 S1 > S3: *p* < 0.001 S2 vs. S3: *p* = 0.19
Wrong findings	303/1336 (22.7%)	365/1060 (34.4%)	730/1536 (47.5%)	*p* < 0.001 S3 > S1: *p* < 0.001 S3 > S2: *p* < 0.001 S1 vs. S2: *p* = 0.25
At least one vessel misinterpreted as nodule in *n* of studies	41/740 (5.5%)	28/740 (3.8%)	136/740 (18.4%)	
Not detected actionable nodules	Altogether 102 missed by at least one tool			
	30/102 (29.4%)	69/102 (67.6%)	54/102 (52.9%)	*p* < 0.001 S1 < S2/S3: *p* < 0.001 S2 > S1/S3: *p* < 0.001
LA diameter (mm)	13.2 ± 7.3	11.4 ± 5.7	11.6 ± 6.4	*p* = 0.41
Solid	23/30 (76.7%)	60/69 (87%)	47/54 (87%)	
Part-solid	1/30 (3.3%)	7/69 (10.1%)	6/54 (11.1%)	
Nonsolid	6/30 (20%)	2/69 (2.9%)	1/54 (1.9%)	S1 > S2/S3: *p* < 0.001
Malignancy risk (Brock model) ≥ 10%	17/30 (56.7%)	29/69 (42%)	22/54 (40.7%)	*p* = 0.32

### Lung nodule evaluation

Each software tool ordered the nodules by long axis (S1, S2) and by a combination of long axis and malignancy risk (S3), respectively. The five largest, actionable nodules from each tool were evaluated in detail regarding density, location, size, visual segmentation quality, and longitudinal consistency, as well as VDT (if available). Solid nodules predominated across all tools (89–91%), with a similar distribution of part-solid and nonsolid types. Nodule size and volume ranged similarly across tools, with no statistically significant differences in long axis, mean diameter, or volume measurements.

Segmentation quality was rated as excellent or satisfactory in most cases, with no significant difference between S1 and S3. S2 did not provide a visible segmentation outline; therefore, segmentation quality could not be assessed.

Growth, shrinkage, or stability rates from the original to a follow-up scan were comparable between the tools. All results are summarized in Table 4.

**Table 4:** Comparison of actionable lung nodules evaluation between S1, S2, and S3

	S1	S2	S3	Comparison
Sum of actionable nodules (up to five per patient)	350	308	331	
Solid	320/350 (91.4%)	275/308 (89.3%)	298/331 (90%)	
Part-solid	25/350 (7.1%)	22/308 (7.1%)	19/331 (5.7%)	
Nonsolid	5/350 (1.4%)	10/308 (3.2%)	12/331 (3.6%)	
Upper-lobe (UL) localization	166/350 (47.4%)	142/308 (46.1%)	173/331 (52.3%)	
Long axis (LA) diameter (mm)	5–30	5–30	5–30	*p* = 0.07
Mean average from long and short axis (mm)	4–26	3–26	4–27	*p* = 0.07
Volume (mm^3^)	15–8470	31–7240	19–9019	*p* = 0.16
Segmentation accuracy				
Excellent	282/350 (80.6%)	na	244/331 (74.4%)	S1 vs. S3: *p* = 0.19
Satisfactory	28/350 (8%)		37/331 (11.3%)	
Poor	26/350 (7.4%)		35/331 (10.7%)	
Failure	14/350 (4%)		12/331 (3.7%)	
Follow-up (FU) available	118/350 (33.7%)	110/308 (35.7%)	118/331 (35.6%)	
FU interval (days)	27–216 (103 ± 46)	10–371 (133 ± 86)	37–375 (128 ± 71)	
Comparison available	100/118 (84.7%)	83/110 (75.5%)	99/118 (83.9%)	
Nodule growth	30/100 (30%)	33/83 (39.3%)	39/99 (39.4%)	
VDT calculated	29/30 (96.7%)	31/33 (93.9%)	33/39 (84.6%)	
VDT range (days)	35–923	97–10,733	34–5898	
VDT median (days)	329	1106	381	
No relevant growth (< 25%)	10/100 (10%)	1/83 (1.2%)	1/99 (1%)	
Nodule shrinkage	53/100 (53%)	45/83 (54.2%)	55/99 (55.5%)	
Calculation not available	7/100 (7%)	5/83 (6%)	4/99 (4%)	

### Hypothetical downstream management assuming incidental detection of pulmonary nodules

#### Fleischner guidelines

Fleischner guidelines were applicable to 98 (28%) of 350 (S1), to 81 (26.3%) of 308 (S2), and to 94 (28.4%) of 331 actionable nodules (S3). A substantial number of nodules needed to be excluded from this analysis due to patients’ underlying malignancies or age criteria that rendered the Fleischner guidelines inapplicable.

No follow-up or optional follow-up (nodules < 6 mm if solid or if subsolid and single) was recommended for 17 nodules detected by S1 (17.3%), 18 by S2 (22.2%), and 10 by S3 (10.6%). A 6–12-month FU (single solid nodules between 6–8 mm, or single ground glass nodules ≥ 6 mm) was recommended for 11 (S1, 11.2%), 12 (S2, 14.8%), and 7 (S3, 7.4%) nodules. A 3–6-month FU (solid nodules > 8 mm, part-solid nodules ≥ 6 mm, and multiple solid or subsolid nodules) was recommended for 44 (S1, 44.9%), 29 (S2, 35.8%), and 50 (S3, 53.2%) nodules. Optional FU at 3 months/PET-CT/biopsy was recommended for 26 (S1, 26.5%), 22 (S2, 27.2%), and 27 (S3, 28.7%) nodules.

**Fig. 4:**
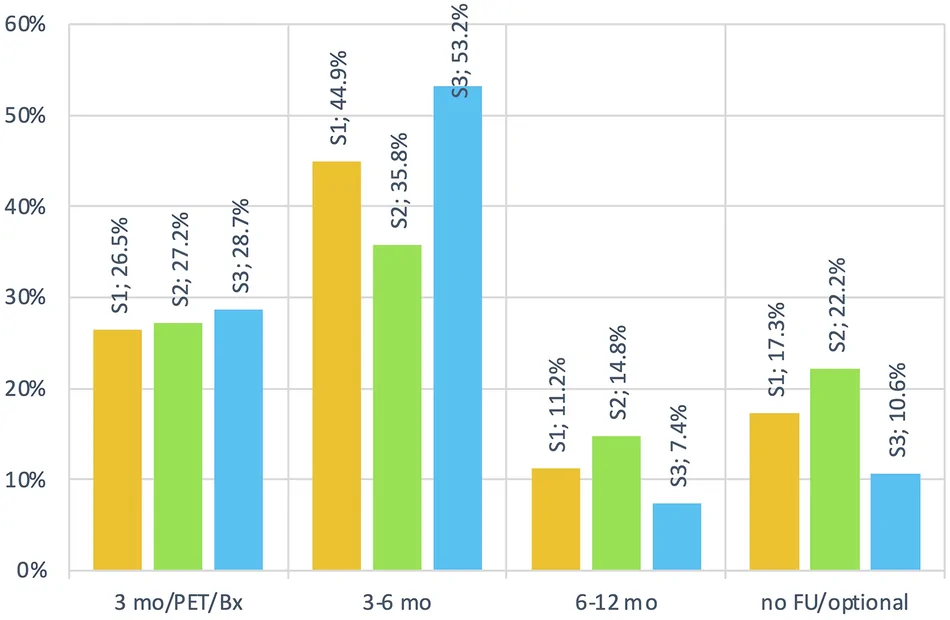
Management procedure using the Fleischner guidelines without significant deviations

The recommendations based on the Fleischner guidelines were statistically independent from the used tool (*p* = 0.19) (Fig. 4).

#### BTS guidelines

BTS guidelines were applicable to all actionable nodules; none needed to be excluded.

A FU at 3 months (solid nodules with a baseline volumetry of 80–300 mm^3^ (6–8 mm)/all subsolid nodules) was recommended for 114 (S1, 32.6%), 93 (S2, 30.3%) and 100 (S3, 30.4%) nodules, FU at 1 year (solid nodules with a baseline long axis of 5–6 mm) for 26 (S1, 7.4%), 44 (S2, 14.3%) and 18 (S3, 5.5%) nodules.

The Brock model was applied to solid nodules with a baseline volume of ≥ 300 mm^3^, (≥ 8 mm) regarding 210 nodules detected by S1 (60%), 170 by S2 (55.2%), and 211 by S3 (64.1%). Nodules with a calculated malignancy risk < 10%, warranting continued surveillance at 3 months, accounted for 102 nodules in S1 (48.6%), 77 in S2 (45.3%), and 92 in S3 (43.6%). Nodules with a risk of ≥ 10%, necessitating additional PET-CT evaluation, comprised 108 nodules in S1 (51.4%), 93 in S2 (54.7%), and 119 in S3 (56.4%).

**Fig. 5:**
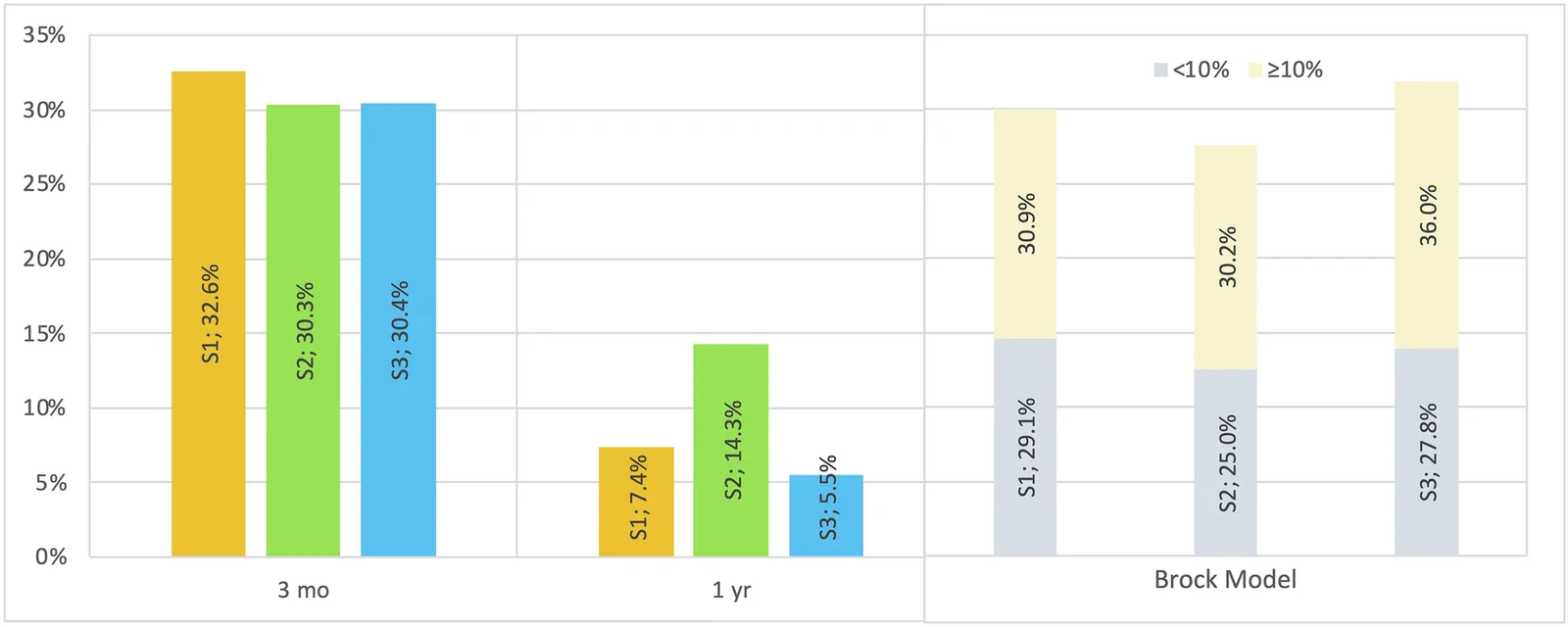
Management procedure using the BTS guidelines showing significant deviation regarding 1-year FU

Application of the BTS guidelines varied significantly depending on the software tool used, with S2 recommending significantly more 1-year follow-ups compared to S3 (*p* < 0.001) (Fig. 5).

There was no significant difference in the results of the Brock evaluation between the tools (*p* = 0.58).

### Evaluation of VDT

The BTS guidelines require the use of VDT for solid nodules to determine management strategy: definitive treatment for nodules with a VDT ≤ 400 days, biopsy for those with VDT between 400–600 days, and cessation of further surveillance for nodules with a VDT > 600 days.

For 29 (S1), 31 (S2), and 33 (S3) actionable nodules, VDT could be established. The intervals between the two examination timepoints were 27–216 days (S1; 103 ± 46 days), 10–371 days (S2; 133 ± 86 days), and 37–375 days (S3; 128 ± 71 days).

The median VDT was 329 days (S1, 95% CI [185, 504], range 35–923 days), 1106 days (S2, 95% CI [772, 1780], range 97–10,733 days), and 381 days (S3, 95% CI [214, 792], range 34–5898 days). VDT was significantly longer in S2 compared to S1 (*p* = 0.001) and S3 (*p* = 0.02).

A VDT of ≤ 400 days was observed in 17 (S1, 58.6%), 7 (S2, 22.6%) and 18 (S3, 54.5%) nodules, a VDT of 400–600 days and of > 600 days in 7 (S1, 24.1%) and 5 (S1, 17.2%), 1 (S2, 3.2%) and 23 (S2, 74.2%) and in 3 (S3, 9.1%) and 12 (S3, 36.4%) nodules.

**Fig. 6:**
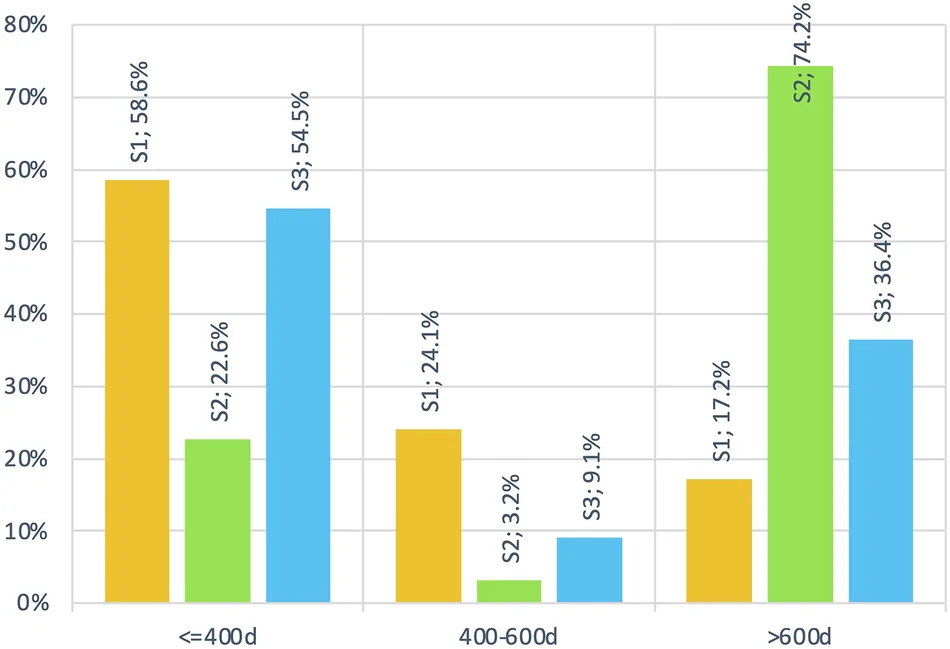
VDT categories showing significant deviations for S1 and S2

We observed a statistically significant difference in the category of VDT (≤ 400 days, 400–600 days, > 600 days) and subsequently a statistically significant difference in the further procedure (*p* < 0.001). Post hoc analysis showed less nodules with a VDT > 600 days (discharge, *p* < 0.001), but more nodules with a VDT between 400 and 600 days (biopsy or FU, *p* = 0.007) for S1 and less nodules with a VDT ≤ 400 days (definitive management, *p* = 0.001), but more nodules with a VDT > 600 days (discharge, *p* < 0.001) in S2 (Fig. 6).

## Discussion

This study compared three commercially available CE‑certified software tools for the detection and segmentation of pulmonary nodules and evaluated their potential impact on hypothetical clinical management of incidental lung nodules when applying both the Fleischner Society recommendations and the British Thoracic Society (BTS) guidelines. This approach was chosen to reflect the real‑world comparative performance of AI‑based software tools routinely applied to thoracic CT examinations in clinical practice. To our knowledge, no comparable multi‑vendor post‑market evaluation has been published to date, despite such investigations being explicitly encouraged by the European Union Artificial Intelligence Act and promoted by the European Society of Radiology (ESR). According to our study aim, we did not intend to provide data on screening using Lung-RADS and other software tools [6], compare human and AI readers [7], or provide data on histopathologic correlation of detected malignant nodules [8].

In our cohort, the three software tools detected a mean of 1.4 to 2.1 nodules per patient, of which 24–32% were “actionable” and required further evaluation. In contrast, published prevalence of lung nodules varies widely, ranging from 0.39% to 27.75% in non-cancer populations and up to 59.3% in lung cancer screening cohorts [9, 10]. Although the total number of detected nodules differed, the detection rates of actionable nodules were comparable between the tools, particularly with respect to wrong and clearly benign lesions. These findings reflect fundamental differences in the underlying algorithms and detection thresholds employed by each vendor. The tested tools generated numbers between 0.9 and 1.6 wrong findings or clearly benign lung nodules per patient, consistent with previous reports indicating that CAD systems commonly produce a substantial number of not actionable findings and an increase in those when radiologists used lung nodule detection software compared to unaided reading [11]. This highlights the inherent trade-off between sensitivity and specificity in CAD systems. In current practice, there is a general tendency to favor higher sensitivity, even at the cost of increased rates of clinically irrelevant findings. Nevertheless, compared with each other, each tool missed between 30 and 69 nodules, which were detected by one or two of the other tools. Importantly, 40% to 57% of those undetected nodules had a calculated malignancy risk exceeding 10% according to the Brock model.

In our cohort, most nodules were solid, 5.7–7.1% were part-solid, and 1.4–3.6% nonsolid. The HANSE LCS trial showed that the sensitivity of software tools for solid nodules was better than for nonsolid nodules [6]. In comparison, the Lung Cancer Action Project using only human readers showed 81% of all nodules to be solid, 7% part-solid, and 12% nonsolid [2]. Software tools demonstrate higher accuracy for solid nodules, whereas subsolid nodules might remain underrepresented [6]. Paramasamy et al reported that 50% of all nodules missed by the CAD were nonsolid, compared to 35.3% solid and 14.7% part-solid nodules [6]. In our study, only the software tool S1 missed more nonsolid than part-solid or solid nodules relative to the total sample. Despite the lower prevalence of part-solid nodules, their accurate detection is critical due to their significantly higher risk of malignancy [2].

Regarding the accuracy of lung nodule segmentation, S1 and S3 showed comparable performance (both about 85% “excellent” or “satisfactory”); S2 could not be determined. Segmentation becomes especially challenging when nodules are located adjacent to complex anatomical structures such as blood vessels, the pleura, or in regions close to atelectasis [12, 13]. In many commercially available software tools—including two of the three evaluated in this study—manual adjustment of the segmentation is not possible, leaving the user reliant on the accuracy of the automated delineation.

The accuracy of segmentation and size measurement is crucial, particularly when guideline-based algorithms are applied [1, 3]. Even small deviations in measured size may result in different FU intervals or even trigger invasive diagnostic procedures [1, 3]. Application of the BTS guidelines revealed statistically significant differences in FU recommendations, particularly with respect to 1-year FUs. The 5–6 mm range, for which 1-year FU is recommended by BTS, is particularly narrow, and even minor measurement discrepancies between tools can lead to substantial changes in clinical management recommendations [1]. The Fleischner guidelines do not include this 5–6 mm category [3], which may explain the absence of significant differences. As our study did not include a screening cohort, direct comparisons to data from studies using Lung-RADS could not be performed [6, 14].

VDT is a key factor affecting management and can considerably differ between tools. The calculation of VDT depends on the assumption that the same lesion is correctly identified in both scans. Errors may lead to misclassification of growth behavior, potentially resulting in inappropriate surveillance intervals or unnecessary diagnostic interventions [1, 3]. S2 systematically produced substantially higher VDT values than the others. Although there were no significant differences in measured nodule volume, even minor discrepancies can lead to substantial differences in calculated VDTs because of the logarithmic nature of the formula [15]. In our study, these variations in VDT had a direct impact on clinical management recommendations, underscoring the sensitivity of VDT-based assessment to subtle computational or segmentation differences. In contrast to our findings, two previous studies identified differences in measured volume as the main driver of divergent management outcomes [6, 14].

## Strengths and limitations

A major strength of our study is the use of three different commercially available tools to evaluate the same examinations. For clinical routine, typically only one tool is available, and direct comparison is rarely possible. Furthermore, we applied these tools to real-world data derived from an intentionally heterogeneous patient cohort and a wide spectrum of examinations, including both patients with and without malignancies, NECTs, CECTs, and CE-PET-CTs. Similar to human readers, the AI tools must handle different imaging modalities and vendors. This reflects the variability encountered in routine clinical practice and thus increases the external validity and generalizability of our post-marketing surveillance.

A major limitation of our study is the lack of a universal ground truth, as we did not include an independent human reader for the entire cohort. Consequently, there is no data on the total number of nodules potentially missed by all three AI tools, preventing an absolute sensitivity analysis. Furthermore, histological correlation for the actionable nodules was not feasible in this routine clinical cohort, as most small incidental nodules do not meet the criteria for invasive biopsy in real-world practice.

Additionally, the different default settings and non-adjustable algorithms of the three tools must be considered. Since the tools did not consistently detect the same nodules or assign identical significance, a direct one-to-one nodule-based comparison was not feasible. Therefore, our analysis focused on the total detection rates and the hypothetical impact on downstream management based on established guidelines, reflecting the practical utility of these tools in a high-throughput clinical setting.

Inter-reader variability was assessed only in a subset (50 patients/265 nodules). However, agreement was high at 94% (*p* = 0.89), facilitated by unambiguous definitions for benign findings and anatomical pitfalls. Post hoc power analysis confirmed that this sample size was sufficient to validate these results.

Finally, our findings reflect the performance of the specific software versions available at the time of analysis. The results presented here may not apply to future, updated versions of the same tools (e.g., the unavailability of outlined segmentation in S2, which in the meantime could be available).

## Conclusion

In this study, three commercially available AI‑based software tools showed comparable performance in detecting and segmenting actionable pulmonary nodules. However, minor measurement variations may significantly impact downstream management when using guidelines with narrow thresholds, such as the BTS criteria. Fleischner Society guidelines appear more robust to these inter-software variations. All systems required human oversight due to missed nodules and, in particular, a high number of false‑positive and clearly benign findings. As all tools identified more wrong or benign lesions than truly actionable nodules, standalone AI‑based reporting carries a substantial risk of overdiagnosis and potential overtreatment.

## Abbreviations

BTS: British Thoracic Society
CAD: Computer-aided detection
CECT: Contrast-enhanced CT
CE-PET-CT: Contrast-enhanced PET-CT
CI: Confidence interval
FU: Follow-up
LA: Long axis
NECT: Non-contrast-enhanced CT
VDT: Volume doubling time
